# Geographic and urban–rural disparities in dietary energy and macronutrient composition among women of childbearing age: findings from the China health and nutrition survey,1991–2015

**DOI:** 10.1186/s12937-023-00851-y

**Published:** 2023-05-09

**Authors:** Jian Zhao, Lijun Zuo, Jian Sun, Chang Su, Huijun Wang

**Affiliations:** 1 Department of Epidemiology and Statistics, Institute of Basic Medical Sciences, School of Basic Medicine, Chinese Academy of Medical Sciences, Peking Union Medical College, Beijing, 100005 China; 2 Aerospace Information Research Institute, Chinese Academy of Sciences, Beijing, 100101 China; 3 School of Public Health and Management, Ningxia Medical University, Yinchuan, 750004 China; 4 National Institute for Nutrition and Health, Chinese Center for Disease Control and Prevention, Beijing, 100050 China

**Keywords:** Women of childbearing age, Urban–rural disparities, Geographic disparities, China

## Abstract

**Background:**

Understanding nutritional status among women of childbearing age (WCA) is of increasing concern, as nutrient intakes may affect the health of WCA and well-being of their offspring. This study aimed to investigate secular trends of dietary energy and macronutrients intakes and access longitudinally the urban-rural and geographic disparities among Chinese WCA.

**Methods:**

A total of 10,219 participants were involved in three rounds of the Chinese Health and Nutrition Survey (CHNS:1991, 2004, and 2015). Average macronutrients intakes were compared against the Chinese Dietary Reference Intakes Standard (DRIs) to better assess adequacy. Mixed effect models were used to estimate the secular trends of dietary intake.

**Results:**

A total of 10,219 participants were involved. Dietary fat, the percentage of energy (%E) from fat, and the proportion with more than 30% of energy from fat and less than 50% from carbohydrates increased notably over time (p < 0.001). In 2015, urban western WCA had the most dietary fat (89.5 g/d), %E from fat (41.4%), with the highest proportion of energy from fat (81.7%) and carbohydrate (72.1%) out the range of DRIs. From 1991 to 2015, the average urban-rural differences in dietary fat decreased from 15.7 g/d to 3.2 g/d among eastern WCA. However, it increased to 16.4 g/d and 6.3 g/d among central and western WCA, respectively.

**Conclusion:**

WCA was experiencing a rapid transformation to a high-fat diet. Temporal variation with obvious urban-rural and geographic disparities in dietary. energy and macronutrient composition persistently existed among Chinese WCA.These findings have implications of future public strategies to strengthen the nutrition propaganda and education of balanced diet for WCA to help them to improve their nutritional status, especially for those living in western China.

## Introduction

Women of childbearing age (WCA) are at greater risk of adverse health outcomes than other groups due to their higher physiological requirements. In particular, the need for appropriate nutrient intakes increases significantly during pregnancy, lactation, or menstruation. Adequate access to nutritional information not only helps to provide targeted dietary advice for WCA but also indirectly promotes the well-being of their children, who will eventually become the main workforce or leaders of the country [1–3]. In China, to better assess the adequacy, the average macronutrient intake is usually compared with the Chinese Dietary Reference Intake Standards (DRIs),which have been used as a criterion for determining the level of recommended dietary intake in Chinese [4]. Meanwhile, according to the DRIs, a balanced diet consisting of 20 to 30% total energy intake from fat,15% total energy intake from protein, and 50 to 65% total energy intake from carbohydrates is critical for promoting maternal health and optimizing fetal development [5, 6]. WCA with an appropriate diet could maintain the birth weight of their offspring within a reasonable range [7]. This is especially true among Chinese women because of the traditional role of mothers in families and communities [8]. However, nutrition problems during the childbearing years are often neglected.

In China, people’s diets and lifestyles have undergone significant changes towards choosing a western-style diet high in fat and animal-based foods with rapid urbanization and socioeconomic transition over the past few decades [9]. In particular, an unbalanced diet potentially leads to an increased risk of future malnutrition and non-communicable diseases (NCDs),especially among vulnerable groups such as WCA [10]. However, the dietary imbalance is often associated with obesity, which also leads to many other health complications [11]. The recent Nutrition and Chronic Disease Surveillance of Chinese residents reported that over 50% of WCA in China are overweight or obese, which poses a major challenge to the health of the population and the medical system [12].

Being the largest developing country in the world, WCA accounted for 24.7% of the total population of China. The Chinese government recently adopted a program encouraging couples to have three children, which has increased the number of multi- parent families [13]. According to the National Bureau of Statistics of China [14], roughly 14.7 million babies were born in 2019. As a result, it’s vital to focus on Chinese WCA’s nutritional status. Furthermore, China covers a large geographic area and has a considerable dual urban-rural economic structure as well as regional development differences [15]. The health status of WCA varies significantly in different geographical regions [16]. Previous findings indeed found significant health gaps in western, central, and eastern China, for example, residents in the southeastern coastal areas tend to have a better health status than those in western China [17]. Nutrition is one of the most important factors causing the health disparities between urban and rural areas in China. Therefore, the nutrient intake of WCA from different geographic locations is an important issue that cannot be neglected.

To date, a growing volume of epidemiological studies has been conducted to evaluate nutrient adequacy and urban-rural disparities in nutrition in China. However, most investigations in this area have been focused on the Chinese elderly and children, but seldom on WCA [18–20]. Furthermore, there is a large variation in study design and methodology, and often small samples are used, resulting in few generalize findings. Hence, the objective of the present study was to access secular trends in dietary energy and macronutrient intake and further ascertain the urban-rural and geographic disparities in a large sample of Chinese WCA.

## Materials and methods

### Study design and subjects

This was a population-based, longitudinal study. The related data of WCA were derived from the China Health and Nutrition Survey (CHNS), which was conducted every two or three years from 1989 up to the present [21]. Each round of the survey, included basic demographic and socioeconomic characteristics, dietary intake, and behavioral factors. A stratified multistage, random-cluster sampling method was used to draw households from urban and rural areas in China [22]. The CHNS has become a crucial source of data to investigate the impact of the social and economic transformation of Chinese society on the health and nutrition status of the Chinese population. Additional details regarding the CHNS are provided elsewhere [23].

Trained healthcare workers conducted face-to-face interviews with participants and their families at baseline to collect data on demographic characteristics, eating habits, and frequency of dietary, and nutrient intake, as well as behavioral factors of WCA. To observe and compare the degree of transformation and trends at intervals of more than a decade, the present analysis was based on three rounds of the CHNS, conducted in 1991, 2004, and 2015. A total of 10,219 women aged between 15 and 49 years old with complete data on demographic, socioeconomic status, and dietary information in a survey year were included in the present study after excluding participants with (n = 121) implausible energy intakes (< 600 kcal or > 4000 kcal) from the analysis [24].

### Dietary data collection

The three consecutive 24-hour dietary recall was used to collect food intake information in each wave of CHNS, except for the condiment intake, which was collected by the weighting method [25]. Participants were required to report the amount (in grams) of various foods and beverages consumed at home and out (during a 24-h period) with food models and picture aids in the household interview. Furthermore, the average intake of dietary energy (kcal/d), fat (g/d), carbohydrate (g/d), and protein (g/d) values were calculated by the Chinese Food Composition Table (CFCT) [26].

### Sociology-demographic data and anthropometrics

The educational level was classified as primary school or illiterate, middle school, high school, or above. The per-capital annual income in each survey was inflated to values in 2015. The marital status was divided into single or married. Participants’ height (seca 260) and weight (seca 880) were measured by trained health workers, and their Body Mass Index (BMI) was calculated using the standard formula: weight in kilograms/square of height in meters (kg/m^2^) [27]. Participants reported all physical activities (PA), and we converted the time spent on each activity into a metabolic equivalent of task (MET) hours per week based on the compendium of physical activities [28]. Smoking and drinking status were classified into non-smokers or smokers and non-drinkers or drinkers. Meanwhile, the influence of urban-rural locations and geographical regions in China (assigned as eastern, central, and western) were also considered [29].

### Statistical analyses

These analyses were performed with the use of SAS software (SAS Institute, USA) version 9.4. Continuous variables were presented as mean and standard error (SE) and categorical variables by percentage and SE of percentage. The chi-square (χ^2^) test was used to assess the association between the different levels (below, meeting, and above the recommendations) of DRIs for macronutrients. T-tests were used to estimate the differences in dietary intake within a year. Mixed effect models were used to calculate the adjusted intake of dietary energy, macronutrients, and macronutrient-energy percentages, and to estimate the dietary trend across the survey rounds and the dietary differences between years, stratified by urban-rural and geographic districts. Meanwhile, dietary energy and macronutrient composition were treated as the dependent variables in each model. Statistical significance was considered if p values＜0.05.

## Results

### Characteristics of the study participants

The general characteristics of participants in three rounds of CHNS (1991, 2004, 2015) are presented in Table 1. This study was conducted on 3730, 2866, and 3623 WCA in 1991, 2004, and 2015, respectively. Marital status, education level, income, BMI, PA, region, district, and drinking behavior showed significant temporal trends across three rounds of surveys (p < 0.001). From 1991 to 2015, urban participants increased from 14.1 to 22.9% and the mean BMI increased from 21.8 kg/m^2^ to 23.5 kg/m^2^, while the mean PA decreased from 462.6 METs/w to 166.6 METs/w. Participants who are married, or have a higher education level became more prevalent over time (p < 0.001). The smoking status of WCA was relatively stable, but the drinking status increased significantly over time (p < 0.001). The proportion of WCA from eastern China increased notably from 1991 to 2015 (p < 0.001).

**Table 1:** General Characteristics of WCA by CHNS Rounds, 1991–20,151−2

Characteristic	1991	2004	2015	P-value
Sample(N)	3730	2866	3623	-
Education level (%)				
Primary school/below	51.6 (0.8)	35.2 (0.9)	18.1 (0.7)	< 0.001
Middle school	32.1(0.8)	36.1(0.9)	37.4(0.9)	
High school/above	16.3 (0.6)	28.7(0.8)	44.5 (0.9))	
Marital status (%)				
Single	23.8(0.7)	14.2(0.7)	11.50.6)	< 0.001
Married	76.2(0.7)	85.8(0.7)	88.5(0.6)	
Income (1k yuan)	3.8 (0.1)	8.9 (0.2)	44.8 (1.4)	< 0.001
BMI (kg/m^2^)	21.8(0.1)	22.8(0.1)	23.5 (0.1)	< 0.001
PA(METs/w)	462.6 (4.6)	246.0(4.0)	166.6(2.8)	< 0.001
Smoking (%)	2.0(0.1)	2.7(0.1)	3.0 (0.1)	0.507
Drinking (%)	12.0 (0.6)	9.3(0.7)	7.2(0.7)	< 0.001
Region (%)				
Urban	14.1(0.6)	13.9(0.6)	22.9 (0.7)	< 0.001
Rural	85.9(0.6)	86.1(0.6)	77.1(0.7)	
District (%)				
Eastern	35.8(0.8)	44.7(0.9)	46.0(1.0)	< 0.001
Central	38.2(0.8)	33.3(0.9)	26.0(0.8)	
Western	26.0(0.7)	22.0(0.8)	28.0(0.9)	

^1^Continuous variables were presented as mean and standard error (SE) and categorical variables by percentage and SE of percentage in parentheses,^2^ There were significant trends in each subgroup over the survey round, except for smoking status

### Trends in energy intake and macro-nutrient composition

Table 2 shows the secular trends of dietary energy and macronutrient composition among Chinese WCA from 1991 to 2015. The dietary fat intake of these participants increased notably from 62.1 g/d to 72.9 g/d over time. However, the average intake of dietary energy, protein, and carbohydrate decreased significantly from 2241.1 kcal/d, 73.8 g/d, and 421.2 g/d in 1991 to 1870.1 kcal/d, 58.5 g/d, and 244.4 g/d in 2015, respectively. Additionally, from fat increased from 24.5 to 34.6%, whereas %E from carbohydrates decreased from 63.1 to 52.5% over time.

**Table 2:** Trends of the Dietary Energy and Macronutrient Composition of WCA by CHNS Rounds,1991-2015^1-4^

Dietary Intake	1991	2004	2015	P-value
Energy(kcal/d)	2241.1(11.0)	2080.9(11.3)	1870.1 (14.2)	< 0.001
Fat(g/d)	62.1 (0.7)	66.6 (0.7)	72.9 (0.9)	< 0.001
Protein(g/d)	73.8(0.4)	63.7(0.4)	58.5(0.4)	< 0.001
Carbohydrate(g/d)	421.2 (2.2)	306.2(1.9)	244.4 (2.2)	< 0.001
Fat(%E)	24.5 (0.2)	28.0 (0.2)	34.6 (0.2)	< 0.001
Carbohydrate(%E)	63.1(0.2)	59.6(0.2)	52.5(0.2)	< 0.001

^1^ Mean percentage and standard error(SE)in parentheses,^2^%E, percentage of dietary energy intake.^3^ Values adjusted for, marital status, education, income, BMI, PA, smoking, drinking, region, and district,^4^ Significant trends in each sub-group across all survey years

As seen in Table 3, the dietary energy, protein, carbohydrate intakes, and %E from carbohydrates showed a significant downward trend while the dietary fat intake and En% from fat showed a notable increasing trend over time, regardless of districts and regions in China (p < 0.001). In 2015, WCA in urban western China had the most dietary fat intake (89.5 g/d) and the most %E from fat (41.4%), while they had the least dietary carbohydrate intake (205.9 g/d) and the least %E from carbohydrate (44.4%). Moreover, WCA in rural western China had the least intake of dietary protein (60.5 g/d) in 2015.

**Table 3:** Trends in the dietary energy and macronutrient composition by region and district from CHNS Round,1991–2015^1−4^

	Overall					Urban					Rural			
	1991	2004	2015	p-trend		1991	2004	2015	p-trend		1991	2004	2015	p-trend
**Eastern**														
Energy(kcal/d)	2529.0(17.2)	2006.0(16.6)	1857.3(11.1)	0.000		2286.6 (21.3)	1951.5(17.1)	1846.7(16.9)	0.000		2563.0(18.3)	2013.1(17.8)	1865.0(24.0)	0.000
Fat(g/d)	62.6(1.0)	68.1(1.1)	71.8(1.1)	0.000		76.4(2.7)	75.8(3.3)	73.6(1.8)	0.000		60.7(1.1)	67.1(1.1)	70.5(1.4)	0.000
Protein(g/d)	75.1(0.6)	66.6(0.6)	64.7(0.7)	0.000		71.8(1.6)	68.0(2.0)	67.4(1.1)	0.000		75.6(0.7)	65.8(0.6)	62.7(0.9)	0.000
Carbohydrate(g/d)	415.7(3.5)	286.2(2.7)	236.9(2.7)	0.000		327.7(4.5)	248.9(2.3)	226.9(3.6)	0.000		428.0 (3.7)	291.1(2.9)	244.2(3.9)	0.000
Fat(%E)	22.1(0.3)	30.7(0.3)	34.2(0.3)	0.000		26.5(0.6)	34.8(1.0)	35.0(0.4)	0.000		21.5(0.3)	30.1(0.4)	33.7(0.4)	0.000
Carbohydrate(%E)	65.4(0.3)	57.0(0.3)	51.4(0.3)	0.000		60.1(0.6)	56.2(0.9)	49.8(0.4)	0.000		66.2(0.3)	57.7(0.4)	52.6(0.4)	0.000
**Central**														
Energy(kcal/d)	2626.7(19.1)	2144.3(20.6)	1964.0(25.7)	0.000		2222.9(28.7)	2106.8(20.3)	1950.3(27.5)	0.000		2703.7(21.2)	2151.0(22.1)	1970.8(20.0)	0.000
Fat(g/d)	61.9(1.2)	66.6(1.3)	79.8(1.7)	0.000		74.0(2.6)	82.7(2.5)	89.3(2.9)	0.000		59.6(1.3)	63.7(1.3)	74.4(2.1)	0.000
Protein(g/d)	75.8(0.6)	66.4(0.8)	62.4(0.9)	0.000		68.3(1.3)	69.0(1.8)	64.3(1.5)	0.000		77.2(0.7)	65.9(0.9)	61.5(1.1)	0.000
Carbohydrate(g/d)	441.3.(3.8)	319.2(3.5)	248.5(4.5)	0.000		320.0(5.0)	269.9(5.8)	218.8(5.4)	0.000		464.4(4.1)	327.9(3.6)	263.3(6.1)	0.000
Fat(%E)	26.0(0.4)	27.1(0.4)	36.5(0.4)	0.000		30.3(0.7)	32.4(1.1)	41.0(0.8)	0.000		25.2(0.4)	26.6(0.5)	34.2(0.6)	0.000
Carbohydrate(%E)	61.8(0.4)	62.8(0.4)	50.5(0.5)	0.000		57.1(0.7)	53.7(1.0)	45.6(0.8)	0.000		62.7(0.4)	64.5(0.5)	52.9(0.6)	0.000
**Western**														
Energy(kcal/d)	2426.0(19.5)	2136.5(22.4)	1916.3(23.6)	0.000		2089.1(17.4)	2003.3(14.4)	1891.1(10.2)	0.000		2482.8(17.8)	2162.5(15.6)	1932.5(20.0)	0.000
Fat(g/d)	61.8(1.0)	67.7(0.9)	85.6(1.0)	0.000		65.2(3.1)	74.1(3.4)	89.5(1.3)	0.000		61.2(1.3)	66.7(1.5)	83.1(2.0)	0.000
Protein(g/d)	68.6(0.6)	63.7(1.0)	62.1(0.9)	0.000		65.5(0.6)	66.8(0.6)	64.6(0.7)	0.000		69.2(0.7)	63.1(1.1)	60.5(1.1)	0.000
Carbohydrate(g/d)	398.2(3.9)	326.7(3.8)	223.5(3.3)	0.000		309.2(3.5)	267.0(2.6)	205.9(3.6)	0.000		413.2(4.2)	338.3(4.1)	235.1(3.6)	0.000
Fat(%E)	25.6(0.4)	28.3(0.5)	39.7(0.4)	0.000		30.8(0.3)	34.7(0.3)	41.4(0.4)	0.000		24.6(0.4)	27.0(0.5)	38.6(0.6)	0.000
Carbohydrate(%E)	61.8(0.4)	60.0(0.5)	47.0(0.4)	0.000		55.4(0.8)	52.2(0.9)	44.4(0.6)	0.000		62.9(0.4)	61.6(0.3)	48.9(0.4)	0.000

^1^ Mean percentage and standard error (SE)in parentheses,^2^%E, percentage of dietary energy intake, ^3^ Values adjusted for, marital status, education, income, BMI, PA, smoking, drinking, ^4^ Significant trends in each sub-group across all survey years

**Fig. 1:**
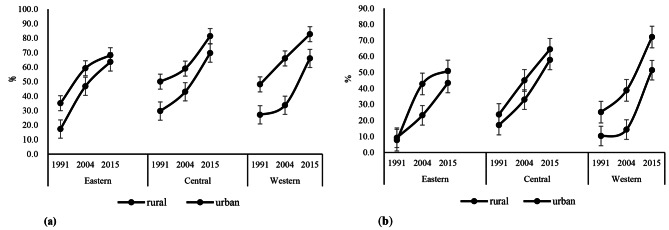
Trends of the proportion of participants with more than 30% of energy from fat and less than 50% of energy from carbohydrate from1991 to 2015 (**a**) The proportion of participants with more than 30% of energy from fat; (**b**) The proportion of participants with less than 50% of energy from carbohydrate

As seen in Fig. 1, the proportion of WCA with more than 30% of energy from fat and less than 50% of energy from carbohydrates increased notably from 1991 to 2015, regardless of location and region (p < 0.001). The average proportion of participants with higher fat intake compared with the DRIs increased from 27.3 to 70.1%. Specifically, the proportion with the higher fat intake increased from 24.5 to 66.7%, and from 44.7 to 75.5% in rural and urban participants, respectively. In addition, the proportion with lower carbohydrate intake increased from 11.7 to 45.8%, and from 18.2 to 60.0% in rural and urban participants, respectively. In 2015, WCA in urban western China had the most proportion who consumed more than 30% of energy from fat (81.7%) and less than 50% of energy from carbohydrates (72.1%).

### Trends of urban-rural disparities

As seen in Table 4, urban-rural disparities in dietary energy and carbohydrate intake of WCA decreased significantly over time across all regions and locations in China (p < 0.001). Generally, WCA from the urban had less dietary energy and carbohydrate intake than their rural counterparts. However, WCA from the rural areas had more dietary protein intake than their urban participants in 1991, while urban participants had more in 2004, and this value increased further in 2015. The average urban-rural disparities in dietary protein intake were 4.0 g/d among WCA in 2015.It is worth noting that the average urban-rural differences in dietary fat in eastern China decreased from 15.7 g/d to 3.2 g/d over time while the differences increased from 14.5 g/d to 16.4 g/d in the central district, and from 4.0 g/d to 6.3 g/d in western China. In 2015, the average urban-rural disparities in dietary fat and carbohydrate intake were 8.1 g/d and 30.5 g/d among WCA, respectively.

**Table 4:** Trends of the Urban-Rural Disparities in the Energy and Macronutrient Composition by region and district from CHNS Round,1991–2015^1−5^

	1991			2004			2015			p-trend
	Urban- rural	P-value		Urban- rural	P-value		Urban- rural	P-value		
**Eastern**										
Energy(kcal/d)	-277.0(4.6)	0.000		-61.6(2.6)	0.000		-18.3(1.6)	0.227		0.000
Fat(g/d)	15.7(3.0)	0.000		8.8(0.6)	0.008		3.2(0.3)	0.162		0.000
Protein(g/d)	-3.8(0.6)	0.000		3.2(0.6)	0.000		4.7(0.5)	0.001		0.000
Carbohydrate(g/d)	-100.3(5.6)	0.000		-42.2(0.4)	0.000		-17.3(1.4)	0.002		0.000
Fat(%E)	5.0(0.6)	0.000		4.7(0.3)	0.000		1.3(0.6)	0.039		0.000
Carbohydrate(%E)	-6.1(0.7)	0.000		-6.5(0.1)	0.000		-2.7(0.6)	0.000		0.000
**Central**										
Energy(kcal/d)	-480.8(1.7)	0.000		-44.2(1.1)	0.000		-20.5(0.5)	0.707		0.000
Fat(g/d)	14.5(0.8)	0.000		16.0(1.3)	0.000		16.4(0.1)	0.000		0.000
Protein(g/d)	-8.9(0.9)	0.000		3.1(0.2)	0.038		3.8(0.1)	0.136		0.000
Carbohydrate(g/d)	-144.4(1.7)	0.000		-58.0(0.8)	0.000		-44.5(0.4)	0.000		0.000
Fat(%E)	5.1(0.76)	0.000		5.8(0.2)	0.000		6.8(0.3)	0.000		0.000
Carbohydrate(%E)	-5.6(0.8)	0.000		-10.8(0.4)	0.000		-7.4(0.7)	0.000		0.000
**Western**										
Energy(kcal/d)	-393.7(2.9)	0.000		-159.2(1.0)	0.009		-41.0(0.8)	0.396		0.000
Fat(g/d)	4.0(0.6)	0.000		5.4(0.4)	0.001		6.3(0.5)	0.028		0.000
Protein(g/d)	-3.7(0.7)	0.036		3.7(0.1)	0.000		4.2(0.1)	0.019		0.000
Carbohydrate(g/d)	-119.9(1.8)	0.000		-71.1(0.7)	0.000		-29.2(0.2)	0.000		0.000
Fat(%E)	6.2(0.6)	0.000		6.7(0.9)	0.000		6.9(0.1)	0.002		0.000
Carbohydrate(%E)	-7.5(0.7)	0.000		-5.4(1.0)	0.000		-4.2(0.2)	0.000		0.000

^1^ Mean percentage and standard error (SE)in parentheses,^2^%E, percentage of dietary energy intake, ^3^ Values adjusted for, marital status, education, income, BMI, PA, smoking, drinking, ^4^ Significant trends in each sub-group across all survey years.^5^ Negative number means urban is smaller than rural

**Fig. 2:**
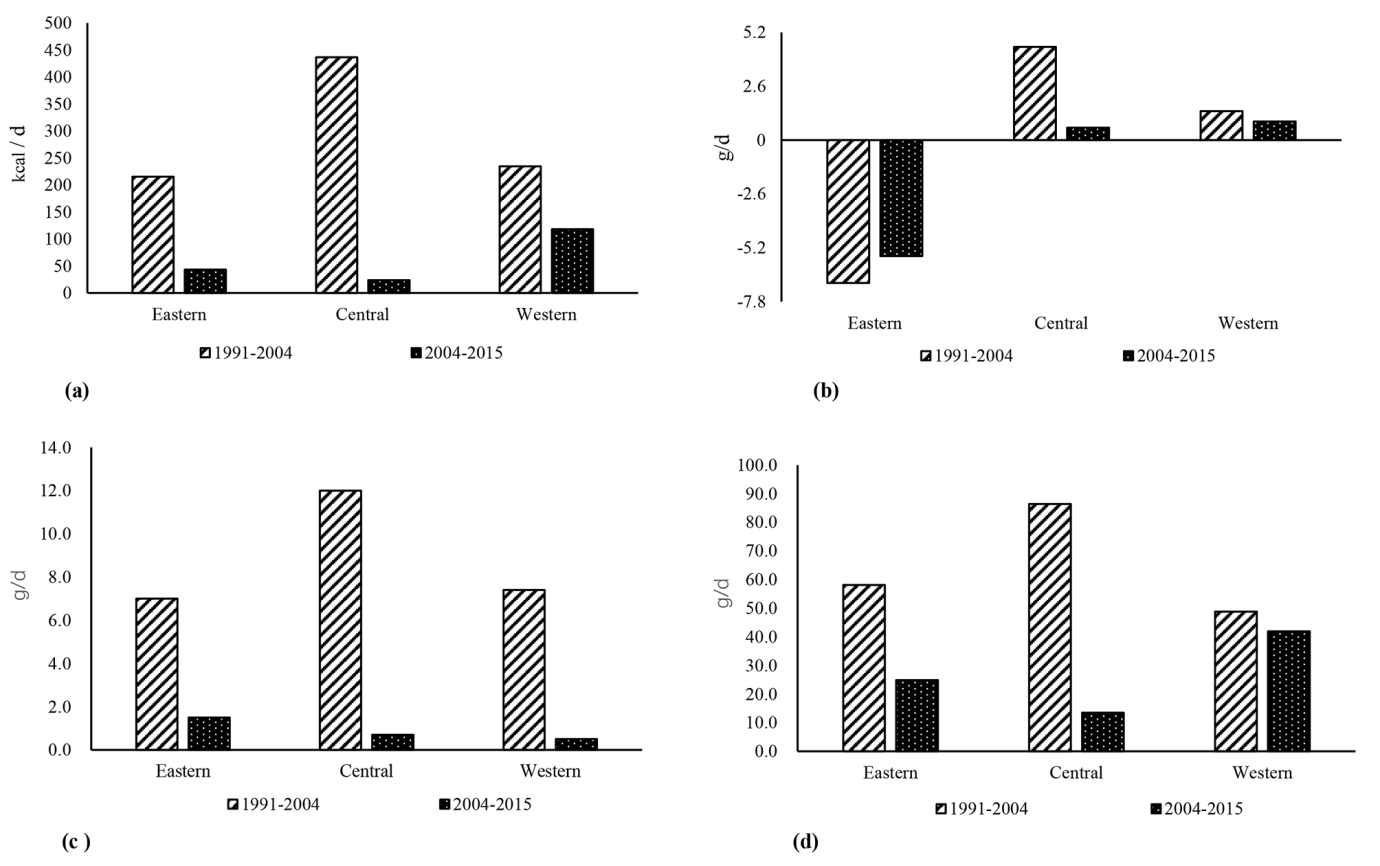
Comparison of the change values of urban-rural differences in each two rounds of surveys. (a) Change values of urban-rural differences in dietary energy intake; **(b)** Change values of urban-rural differences in dietary fat intake; (c)Change values of urban-rural differences in dietary protein intake; **(d)** Change values of urban-rural differences in dietary carbohydrate intake

A Comparison of the change values of urban-rural differences in dietary fat, protein, and carbohydrate intake in every two rounds of surveys is presented in Fig. 2. Generally, the variation in dietary fat, protein, and carbohydrate intake between urban and rural areas was greater in the former two rounds (2004 vs. 1991) than in the latter two rounds (2015 vs. 2004) (p < 0.001). Specifically, the variation of dietary energy intake decreased from 215.4 kcal/d to 43.3 kcal/d, from 436.5 kcal/d to 23.7 kcal/d, and from 234.5 kcal/d to 118.2 kcal/d in the eastern, central, and western districts, respectively. Moreover, the variation of fat intake decreased from − 6.9 g/d, 4.5 g/d and 1.4 g/d to -5.6 g/d,0.4 g/d and 0.9 g/d, protein intake decreased from 7.0 g/d,12.0 g/d and 7.4 g/d to 1.5 g/d,0.7 g/d and 0.5 g/d, and carbohydrate intake decreased from 58.1 g/d, 86.4 g/d and 48.8 g/d to 24.9 g/d,13.5 g/d and 41.9 g/d in the above three areas, respectively.

## Discussion

Based on more than two decades of data from the CHNS, the current study provides useful information on the nutritional status of Chinese WCA. This is the first attempt, to our knowledge, to gain access to the nutrition of Chinese WCA, with a focus on urban-rural and geographic differences, through the interpretation of a longitudinal study on nutritional status. China has achieved significant progress in WCA nutrition. We did notice, however, that Chinese WCA were making a rapid nutritional shift to a high-fat diet over time. Meanwhile, in the distribution of dietary energy consumption and macronutrient composition, there were persisting urban-rural and geographic differences. In comparison to other groups, WCA in urban western China consumes higher dietary fat.

Over the last two decades, China has experienced fast economic expansion and significant urbanization. In the meantime, the Chinese people are undergoing a significant nutritional shift. The current study found that the amount of dietary energy consumed by Chinese WCA decreased over time. In 2015, the average daily energy intake was 1,870 kcal, which was higher than the Moroccan study but lower than the Tunisian women’s consumption [30, 31]. This study indicated that dietary fat intake and the proportion of people who get more than 30% of their energy from fat increased significantly, which is comparable with earlier Chinese studies in various age groups [32]. Excessive intake of fat may lead to obesity, which is also a risk factor for maternal and neonatal mortality. There is growing evidence that the prevalence of obesity among women in China has risen sharply over the past few decades, reflecting the need for more measures to achieve and maintain a healthy weight [33]. Furthermore, PA behavior influences the health and well-being of WCA and their children [34]. Our study found that the BMI of participants showed a significant upward trend, while PA showed a notable downward trend, which is generally consistent with the research trend found in children and the elderly in China [24]. These results indicated that with economic development and social transition, WCA has transformed their lifestyle by increasing unhealthy eating and sedentary behaviors, resulting in a sharp increase in overweight and obesity in China, which is consistent with findings from Latin American women [1].

A previous study reported that commercial logistics facilities in rural areas remain weak and vulnerable, which hampers rural consumption. The food consumers of urban residents mainly rely on market supply, while the rural population mainly relies on self-produced agricultural products [35]. In the present study, urban WCA participants were found to have more dietary fat than rural participants. Consistent with our findings, a previous Brazil cohort study reported that urban/highly industrialized women were more likely to have unhealthy eating habits [36]. In Chinese WCA, there were still obvious geographic disparities with temporal variation in macronutrient intake. In 2015, WCA in urban western China had the highest dietary fat intake, with the highest proportion of energy from fat (81.7%) and carbohydrates (72.1%) beyond the range of DRI. Meanwhile, WCA from rural western China had the least dietary protein intake in the country. This may be since living in western regions, especially in rural areas, with relatively limited resources, complex terrain, and different cultural and dietary traditions, all leads to the complexity of providing adequate health care and affordable nutritious food [37].

China has a remarkable urban-rural dual economic structure, as well as regional differences in economic structure. To narrow the gaps between regions and promote balanced and coordinated development between urban and rural areas, the Chinese government has promulgated a series of policies and measures [38]. A recent preliminary evaluation study on the Rural Primary Health Care (RPHC) program in China pointed out that western China’s RPHC has proceeded well, but remains weaker than that of eastern and central China [39]. Specifically, western regions had the highest under-5 mortality rates and neonatal mortality rates compared with the eastern and central regions of China in 2015 [40]. Despite remarkable progress in maternal survival in China, substantial disparities remain, especially for the poor, less educated, and ethnic minority groups in remote areas in western China [41]. Meanwhile, economic conditions and health services in the western regions are relatively less underdeveloped than in other regions [42]. Further research on the causes of unequal nutrient intake across the socioeconomic context is warranted to provide scientific triage information for policymakers that could improve the nutritional status of WCA and provide an adequate environment for the developing fetus.

Nutritional knowledge and attitudes are important factors in dietary habits. They are essential for good pregnancy outcomes and for improving the nutritional status of their children [43]. Findings from the UK and Ireland indicated that the nutrition knowledge and skills of WCA have a positive impact on women’s nutritional outcomes [44]. Nutrition-related education generally seeks to increase nutritional knowledge and attitude, thereby influencing dietary practices towards a healthier dietary pattern. Our research reported that despite the increased proportion of higher educated WCA, however, the proportion of unhealthy diets has not decreased over time. This suggests the need to provide targeted guidance on dietary knowledge education for Chinese WCA.A previous study found that WCA has a low level of nutritional knowledge and a positive attitude toward receiving nutritional knowledge, but they practice a healthy diet infrequently [45]. These findings have implications for future public strategies to strengthen nutrition propaganda and education on a balanced diet for WCA to help them improve their nutritional status, especially for those living in western China.

Although the present research is significant and novel in some respects, certain limitations should be considered when interpreting the results. First, our findings were based on three consecutive 24-hour dietary recall methods, and the associated over or underestimation of dietary energy and macronutrient intakes is a common situation with such dietary assessment tools. Second, dietary energy and macronutrients existed in a variety of foods, but this study did not estimate the specific types of food consumption by food groups, which makes it impossible for us to consider which food causes the above unreasonable diet, to further provide suggestions on food frequency for Chinese WCA.

## Conclusions

The Chinese WCA was undergoing a dramatic nutritional transformation to an unhealthy, high-fat diet. In the distribution of dietary energy intake and macronutrient composition in the Chinese WCA, obvious urban-rural and geographic disparities persisted. Policymakers should adopt more targeted policies and dietary improvement approaches in urban and rural areas and between different regions of China, as well as strengthen nutrition promotion and education on a balanced diet, especially for those living in western China.

## Data Availability

The datasets generated during and/or analyzed during the current study are not publicly available but are available from the corresponding author on reasonable request and approval from the ethics committee.

## Abbreviations

WCA: women of childbearing age
CHNS: China Health and Nutrition Survey
DRIs: the Chinese Dietary Reference Intake Standards
NCDs: Non-communicable Disease
CFCT: the Chinese Food Composition Table
Midbody: Mass Index
PA: Physical Activity
MET: Metabolic Equivalent of Task
